# Integrated Lipidomics and Proteomics Point to Early Blood-Based Changes in Childhood Preceding Later Development of Psychotic Experiences: Evidence From the Avon Longitudinal Study of Parents and Children

**DOI:** 10.1016/j.biopsych.2019.01.018

**Published:** 2019-07-01

**Authors:** Francisco Madrid-Gambin, Melanie Föcking, Sophie Sabherwal, Meike Heurich, Jane A. English, Aoife O’Gorman, Tommi Suvitaival, Linda Ahonen, Mary Cannon, Glyn Lewis, Ismo Mattila, Caitriona Scaife, Sean Madden, Tuulia Hyötyläinen, Matej Orešič, Stanley Zammit, Gerard Cagney, David R. Cotter, Lorraine Brennan

**Affiliations:** a Department of Psychiatry, Royal College of Surgeons in Ireland, Beaumont Hospital, Dublin, Ireland; b Institute of Food and Health, UCD School of Agriculture and Food Science, Dublin, Ireland; c Conway Institute, UCD School of Biomolecular and Biomedical Science, University College Dublin, Dublin, Ireland; d School of Pharmacy and Pharmaceutical Sciences, Cardiff University, Cardiff, United Kingdom; e MRC Centre for Neuropsychiatric Genetics and Genomics, Cardiff University, Cardiff, United Kingdom; f Faculty of Brain Sciences, Division of Psychiatry, University College London, London, United Kingdom; g Centre for Academic Mental Health, Bristol Medical School, University of Bristol, Bristol, United Kingdom; h Steno Diabetes Center Copenhagen, Gentofte, Denmark; i Department of Chemistry, Örebro University, Örebro, Sweden; j School of Medical Sciences, Örebro University, Örebro, Sweden; k Turku Centre for Biotechnology, University of Turku and Åbo Akademi University, Turku, Finland

**Keywords:** ALSPAC, Early life, Integration, Lipidomics, Proteomics, Psychotic episode

## Abstract

**Background:**

The identification of early biomarkers of psychotic experiences (PEs) is of interest because early diagnosis and treatment of those at risk of future disorder is associated with improved outcomes. The current study investigated early lipidomic and coagulation pathway protein signatures of later PEs in subjects from the Avon Longitudinal Study of Parents and Children cohort.

**Methods:**

Plasma of 115 children (12 years of age) who were first identified as experiencing PEs at 18 years of age (48 cases and 67 controls) were assessed through integrated and targeted lipidomics and semitargeted proteomics approaches. We assessed the lipids, lysophosphatidylcholines (*n =* 11) and phosphatidylcholines (*n =* 61), and the protein members of the coagulation pathway (*n =* 22) and integrated these data with complement pathway protein data already available on these subjects.

**Results:**

Twelve phosphatidylcholines, four lysophosphatidylcholines, and the coagulation protein plasminogen were altered between the control and PEs groups after correction for multiple comparisons. Lipidomic and proteomic datasets were integrated into a multivariate network displaying a strong relationship between most lipids that were significantly associated with PEs and plasminogen. Finally, an unsupervised clustering approach identified four different clusters, with one of the clusters presenting the highest case-control ratio (*p <* .01) and associated with a higher concentration of smaller low-density lipoprotein cholesterol particles.

**Conclusions:**

Our findings indicate that the lipidome and proteome of subjects who report PEs at 18 years of age are already altered at 12 years of age, indicating that metabolic dysregulation may contribute to an early vulnerability to PEs and suggesting crosstalk between these lysophosphatidylcholines, phosphatidylcholines, and coagulation and complement proteins.

SEE COMMENTARY ON PAGE 2

The early identification and treatment of subjects with psychiatric disorders, both psychotic and affective, significantly improves their clinical outcome (1). Consequently, over the last decade, there has been a shift in research focus to a high-risk paradigm for individuals at increased risk for later psychotic disorder (PD) 2, 3, 4. Research over the past 15 years has revealed that 8% to 17% of children and adolescents (5) and 7% of adults (6) in the general population report psychotic experiences (PEs). It is known that these individuals who report subclinical symptoms in early life are at increased risk of PD 7, 8 as well as other disorders 9, 10.

The identification of a biological signature of psychotic illnesses can provide insights into pathophysiological basis of the disorders 11, 12 and also has the potential to be used as a part of biomarker signature for early detection and diagnosis (13). Recent research on schizophrenia and related psychoses has highlighted a number of metabolic perturbations such as glucoregulatory processes 14, 15, lipid metabolism 16, 17, 18, mitochondrial function (19), proline (13), and tryptophan metabolism (20), with the most consistent findings involving pathways common to fatty acids and the pro-oxidant/antioxidant balance 21, 22, 23. A recent systematic review of metabolite biomarkers for schizophrenia by Davison *et al.* (24) revealed that although definite consistencies have been described in the literature, none of the potential biomarkers have been validated reproducibly in large cohorts. Essential polyunsaturated fatty acids, lipid-peroxidation metabolites, phosphatidylcholines (PCs) and lysophosphatidylcholines (LPCs), glutamate, 3-methoxy-4-hydroxyphenylglycol, and vitamin E emerged from this review as potential biomarkers (24), emphasizing the hypotheses of oxidative stress and inflammation (25) and membrane phospholipid alterations (26). While these studies have contributed to our understanding of the disease mechanisms, they generally focus on the adult population that has already transitioned to psychosis, with a majority being medicated. These studies are therefore limited in terms of identifying early molecular signatures of the disease.

To address this issue, we recently applied broad metabolomics, lipidomics, and shotgun and semitargeted proteomics approaches to plasma samples from children at 12 years of age who were reported to develop PD at 18 years of age, from the Avon Longitudinal Study of Parents and Children (ALSPAC) cohort (27). We observed increased PCs and LPCs, and complement and coagulation proteins among these subjects during childhood 22, 23. These findings provided intriguing support for the view that psychosis is associated with a broad range of inflammatory 23, 28, glucoregulatory (29), and lipid (22) dysregulation from early childhood. The interrelationship between these early lipid and protein changes has not yet been investigated. In the current investigation, we have extended our previous work by testing the hypothesis that altered LPCs and PCs and the family of coagulation proteins are associated with not only outcomes of PD, but also the milder phenotype of PEs. Specifically, lipidomic and semitargeted proteomic approaches were employed to semitarget PCs and LPCs and coagulation proteins at 12 years of age among apparently well subjects who go on to develop PEs at 18 years of age in the ALSPAC cohort. These data were then integrated with other complement protein data available of the same subjects to assess the broader functional relationships between these proteins and lipids at 12 years of age among those who later report PEs at 18 years of age.

## Methods and Materials

### Study Cohort

The study comprised subjects from the ALSPAC cohort. The ALSPAC cohort is a prospective general population cohort that includes 14,062 live births from southwest England 30, 31. Written informed consent was acquired before taking the plasma samples. Ethical approval for the study was obtained from the ALSPAC Ethics and Law Committee and the Local Research Ethics Committees (RCSI REC 1240). The study website contains details of all the data that is available through a fully searchable data dictionary (http://www.bristol.ac.uk/alspac/researchers/our-data/).

### Measures of PEs and Comorbid Depression

PEs were identified at 12 and 18 years of age through the face-to-face, semistructured Psychosis-Like Symptoms interview (27), conducted by trained psychology graduates in assessment clinics, and were coded according to the definitions and rating rules for the Schedules for Clinical Assessment in Neuropsychiatry, Version 2.0 (32). Interviewers rated PEs as not present, suspected, or definitely psychotic. Patients were also assessed for the presence of depressive disorder according to the ratings on the Clinical Interview Schedule–Revised whereby Clinical Interview Schedule–Revised scores >7 are defined as fulfilling criteria for depression (28).

### Study Design

We undertook a nested case-control study of the ALSPAC cohort and chose to assess all available plasma samples from 12-year-old children with outcomes of definite PEs at 18 years of age but who did not have PD (27). Available plasma samples from controls of age-matched individuals were then randomly selected. The present study consisted of a hypothesis-driven lipidomic and proteomic analysis of samples from 48 children without suspected or definite PEs at 12 years of age but with definite PEs at 18 years of age (*n =* 48). Control samples (*n =* 67) without suspected or definite PEs at 12 and 18 years of age were selected (see Table 1). Socioeconomic status and presence of depression according to Clinical Interview Schedule–Revised scores were also tested.

**Table 1:** Descriptive Data of the ALSPAC Individuals Included in the Study

	Cases	Controls	*p*
Participants, *n*	48	67	
Male/Female, *n*	22/26	39/28	.19
BMI, kg/m^2^, Mean ± SD	18.16 ± 2.85	17.73 ± 2.53	.40

Descriptive information was compared between cases and controls. Statistical comparisons are from Pearson chi-square or Student’s *t* test as appropriate.

ALSPAC, Avon Longitudinal Study of Parents and Children; BMI, body mass index.

### Plasma Sampling

Nonfasting blood samples were collected from the participants into heparin S-Monovette tubes (Sarstedt, Nümbrecht, Germany). Once collected, samples were stored on ice for a maximum of 90 minutes until processed. Postcentrifugation, the samples were stored at −80°C until further analyses.

### Lipidomic Analysis and Data Preprocessing

Sample processing, data acquisition, and quantification of lipids were performed as previously described (22). Lipidomic analysis was performed using an ultra-high-performance liquid chromatography quadrupole time-of-flight mass spectrometry system (Agilent Technologies, Santa Clara, CA).

Lipidomic data were first processed using MZmine 2 (33), then normalized by lipid-class specific internal standards, and finally quantified using the inverse-weighted linear model (see Supplement). Analysis of lipidomics data was focused on detected PCs (*n =* 61) and LPCs (*n =* 11) based on our previous findings (22).

### Proteomic Analysis and Data Preprocessing

Sample analysis and data acquisition proteins were performed in the same individuals as examined in the current lipidomic analysis and using methods as previously described (23). To improve the dynamic range for proteomic analysis, 40 μL of plasma from each case in all samples was immunodepleted of the 14 most abundant proteins (34) (see Supplement).

Protein digestion and peptide purification was performed as previously described (35) and is further detailed in the Supplement. We used the semitargeted approach of data independent acquisition (DIA) to specifically target 22 members of the coagulation pathway (see Supplemental Table S1). For DIA analysis, 5 μL of each sample was injected into the Thermo Scientific Q-Exactive, connected to a Dionex Ultimate 3000 (RSLCnano; Thermo Fisher Scientific, Bremen, Germany) chromatography system, and data were acquired in DIA mode (see Supplement).

### Statistical Analysis

To assess differences of demographic data among groups, Pearson chi-square test and independent Student’s *t* test were used on categorical and continuous variables, respectively.

#### Early PEs Signatures at 12 Years of Age

Principal component analysis was used on the log-transformed, mean-centered, and scaled-to-unit-variance lipidomics dataset to acquire an overview of the data. For supervised data analysis, uni- and multivariate approaches were performed.

For univariate analysis, the Mann-Whitney *U* test was applied to the untransformed dataset to examine changes of lipids and proteins as related to PEs. Benjamini-Hochberg false discovery rate was applied to account for multiple comparisons.

Multivariate modeling of PEs was performed on the log-transformed data using a partial least squares discriminant analysis of lipidomic profiles with the KODAMA R package v 1.4 (36). Modeling was performed in a repeated double cross-validation framework (37). The goodness of fit and prediction parameters were defined using a standard description reported elsewhere (38). The features were ranked in ascending order based on the absolute loading scores (termed as loading rank) (39). Model performance was further assessed through permutation testing (*R*^2^), considering a statistical significance at *p <* .05.

#### Lipidomics and Proteomics Integration

Regularized canonical correlation analysis was performed on all individuals as an integrative multivariate approach to assess correlations between both lipidomics and proteomics data using the mixOmics R package v 5.2.0 (40).

The method allows the study of the relationship of two multivariate datasets, for instance, the relationship between specific lipids and proteins within the same individuals (41). Quantitative data, derived from DIA analysis, on the broad family of complement pathway proteins were also available on these same subjects (42), and these data were available for integrative analysis. Regularization parameters were estimated by means of a leave-one-out cross-validation. Once the regularized canonical correlation analysis was acquired, the corresponding clustered heat maps, termed clustered image maps, and the integrative network were acquired (43). Data were then exported to Gephi 0.9.2 (44), and the layout algorithm Yifan Hu was used to allow the biological interpretation (45). The network graph describes connections between lipids and proteins based on a similarity score >.3 (45). To evaluate obtained multivariate correlations, a further Spearman correlation analysis was implemented for each variable individually, considering the significant correlation at a *p* value of <.05.

#### Identification of Metabolic Phenotypes

The unsupervised algorithm based on knowledge discovery by accuracy maximization (KODAMA) (46) was used to identify the underlying patterns representative of different metabolic phenotypes across all individuals. This learning algorithm allows an unsupervised clustering of individuals from noisy high-dimensional datasets (36). The partition around medoids method (47) along with a silhouette algorithm (48) were carried out on KODAMA scores to identify the optimal distribution of clusters (49). Further descriptions of this method are shown elsewhere 36, 49. The demographic data and cholesterol profile were then tested among the identified clusters using the K-test. This method predicts an independent variable using the variance in the KODAMA scores by means of permutation testing 49, 50. Thus, causality of phenotyping was explored by other variables (49) such as the cholesterol profile and demographics. Data on cholesterol profile including cholesterol esters and lipoprotein particle data of selected individuals at 7 years of age were measured and reported elsewhere 30, 51. Statistical significance was considered at a false discovery rate–corrected *p* value of < .05.

All statistical analyses were performed in the statistical programming environment R version 3.3.1 (R Foundation for Statistical Computing, Vienna, Austria). Data used for this article will be made available on request to the ALSPAC Executive Committee (alspac-exec@bristol.ac.uk).

## Results

The lipidomic dataset that was used to investigate potential biomarkers of PEs in children 12 years of age who reported PEs at 18 years of age included 61 PCs and 11 LPCs. PCs and LPCs were the focus because of previous results showing a potential lipidomic signature of PD with elevated levels of PCs and LPCs (22). The proteomic dataset that we assessed contained 22 members of the coagulation pathway (Supplemental Table S1) as defined by KEGG pathway analysis (http://www.genome.jp/kegg/pathway.html).

There were no significant differences between the control group and the PEs group in terms of gender, body mass index (BMI), or social class (data not shown). As expected, there was an excess of depression cases among those with PEs compared with controls, with 9 subjects in the PEs group reaching criteria for depression and no cases in the normal control group. Variance in the lipid profiles of individuals was first explored using principal component analysis. No grouping could be observed through principal component analysis when examining factors such as PEs, gender, and BMI.

### Early PEs Signatures at 12 Years of Age

Univariate analysis revealed that a total of 34 molecular lipids and 3 coagulation proteins (plasminogen [PLG], coagulation factor XI, alpha2-antiplasmin) were different between PEs and healthy controls at the nominal *p <* .05 level (Table 2). After false discovery rate correction, 16 lipids and one protein (PLG) remained significantly increased. For multivariate analysis, partial least squares discriminant analysis entailed a resulting model (*R*^2^*Y* = .3) with a permutation test *p <* .05. Interestingly, there is a strong agreement between uni- and multivariate analyses performed individually, in which the lowest *p* values matched the highest loading scores and, thus, lowest loading rank. Significant changes of PCs and LPCs with *p* value and loading rank corresponding to uni- and multivariate analyses, respectively, are also presented in Table 2.

**Table 2:** Differential Plasma Lipids and Proteins Between the Control and PEs Groups

Compound	Control Group	PEs Group	*p*	FDR	LR
Lipid					
PC(34:1)	2571.91	3013.09	.0002	.0066	1
PC(34:2)^a^	3759.47	4303.88	.0002	.0066	2
PC(32:1)	238.88	319.25	.0011	.0161	3
PC(36:4)^a^	135.46	160.55	.0023	.0241	4
PC(36:2)	2940.24	3421.47	.0003	.0067	5
LPC(16:1)	38.27	41.69	.0080	.0361	6
LPC(18:1)^a^	231.84	273.67	.0029	.0259	7
LPC(20:3)^a^	37.21	41.58	.0050	.0259	8
PC(36:1)	721.67	945.44	.0008	.0137	10
LPC(18:2)^a^	394.75	486.68	.0045	.0259	11
PC(38:2)	70.50	86.11	.0023	.0241	12
PC(O-38:6)	28.13	33.58	.0037	.0259	14
PC(38:3)	616.10	752.18	.0079	.0361	15
PC(30:0)	56.88	73.01	.0098	.0414	16
PC(32:0)	175.51	204.39	.0041	.0259	17
PC(36:3)	1753.26	2059.53	.0049	.0259	23
Protein					
PLG^b^	843,597,014.93	1,052,478,260.87	.0006	.0138	–
F11	16,925,970.15	19,053,478.26	.0304	.2379	–
SERPINF2	487,134,328.36	542,565,217.39	.0324	.2379	–

The *p* value of the Mann-Whitney *U* test and loading rank of double cross-validation partial least squares discriminant analysis are shown.

F11, coagulation factor XI; FDR, false discovery rate; LPC, lysophosphatidylcholine; LR, loading rank; PC, phosphatidylcholine; PD, psychotic disorder; PEs, psychotic experiences; PLG, plasminogen; SERPINF2, alpha2-antiplasmin.

a Increased compounds in agreement with O’Gorman *et al*. (22) including PD individuals.

b Increased compounds in agreement with English *et al*. (23) including PD individuals.

### Lipidomics and Proteomics Integration

**Figure 1:**
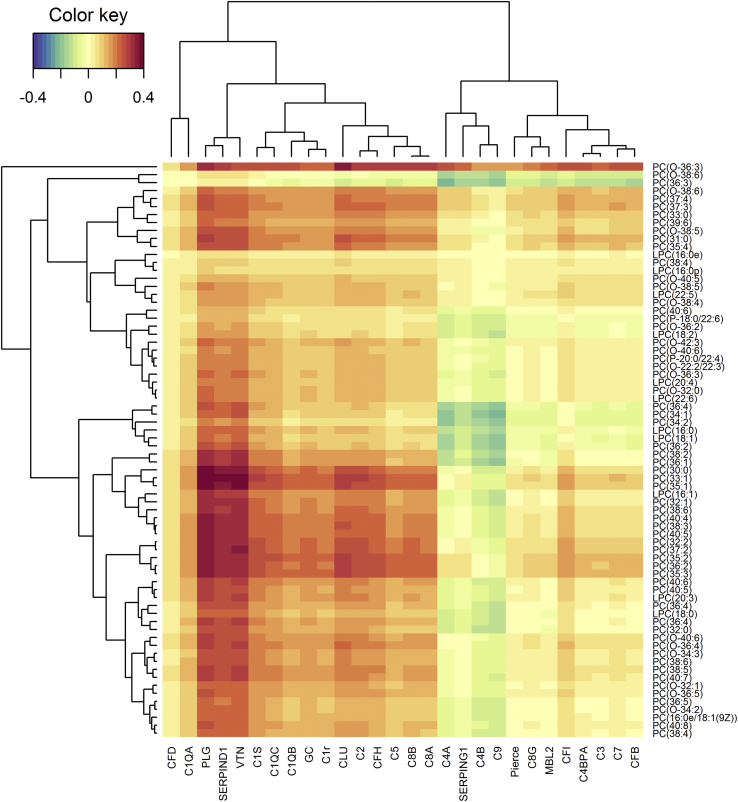
Heatmap analysis performed by using regularized canonical correlations analysis showing the relation between proteomic and lipidomic datasets. For proteomic data, the gene names are displayed. Correlation strengths are indicated by the color key.

**Figure 2:**
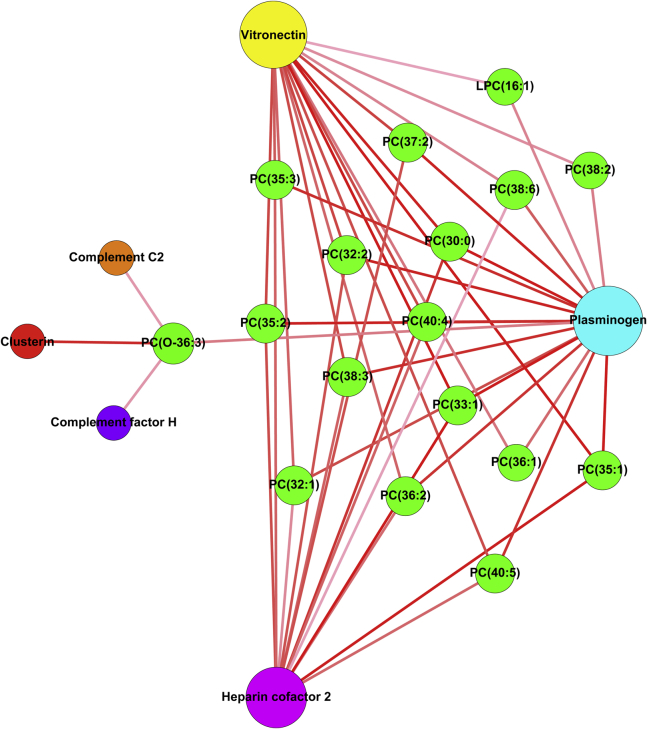
Relevance network graph depicting correlations derived from regularized canonical correlation analysis between lipids and proteins based on a similarity score >.3 (45). Nodes (circles) represent variables and are sized according to number of connections. Lines are colored according to association score with augmented intensity indicating higher correlation scores. LPC, lysophosphatidylcholine; PC, phosphatidylcholine.

The coagulation and complement pathway proteins are closely functionally related. For this reason, we included in our integrative analysis of lipids and proteins the levels of complement proteins in the total dataset for which there were data available (42). The regularized canonical correlation analysis revealed that 17 lipids have a positive correlation with six proteins (PLG, heparin cofactor 2, complement C2, complement factor H, clusterin, and vitronectin), which exceeded a similarity score higher than 0.3. A strong positive relationship with the 16 lipids was observed for coagulation proteins PLG, heparin cofactor 2, and the complement pathway protein vitronectin (Figure 1). A relevance network graph illustrates other minor connections observed for complement proteins clusterin, complement C2, and complement factor H (Figure 2). Interestingly, PLG had the highest number of connections, followed by vitronectin and heparin cofactor 2. Table 3 shows specific lipid connections with PLG, with 10 lipids showing a correlation exceeding a similarity score higher than 0.3.

**Table 3:** Significant Lipids Correlated With Plasminogen From Multi- and Univariate Approaches on the PEs Dataset

Lipid	rCCA	Spearman Correlation	*p*
PC(30:0)^a^	.38	.27	.005
PC(32:0)^a^	.26	.19	.043
PC(34:1)^a^	.24	.26	.006
PC(40:6)	.29	.19	.047
PC(32:1)^a^	.33	.28	.003
PC(38:2)^a^	.31	.20	.039
PC(38:3)^a^	.35	.22	.019
PC(36:1)^a^	.32	.22	.022
PC(35:1)	.39	.25	.007
PC(36:4)^a^	.28	.23	.014
LPC(16:1)^a^	.31	.24	.010
PC(40:5)	.35	.27	.004
PC(40:4)	.35	.26	.006
PC(33:1)	.40	.34	.001
PC(37:4)	.24	.20	.032
PC(36:3)^a^	.22	.19	.043
PC(O-36:3)	.31	.24	.013
PC(31:0)	.28	.21	.029

The *p* values of Spearman correlation analysis are shown. Results are listed for the 18 significant compounds using a *p* value < .05.

LPC, lysophosphatidylcholine; PC, phosphatidylcholine; PEs, psychotic experiences; rCCA, regularized canonical correlation analysis.

a Significant lipids associated with PEs development in the present study.

### Underlying Clustering in the Data

**Figure 3:**
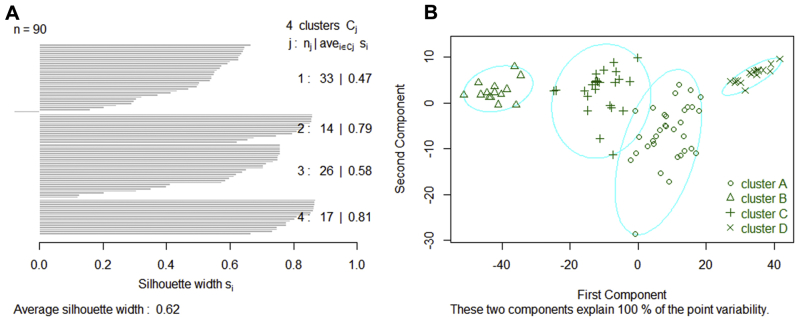
Partition around medoids analysis of the knowledge discovery by accuracy maximization output: **(A)** silhouette plot of partition around medoids including the optimal number of clusters (j), individuals at each cluster (n_j_), and the average silhouette width by samples (ave_i∊Cj_ S_i_); **(B)** clustering according to the calculated silhouette mean values.

To detect potential underlying metabolic phenotypes present in the study population, the KODAMA algorithm was applied to all individuals with available clinical data (*n =* 90). Following this, partition around medoids clustering was performed on KODAMA scores to identify underlying similar phenotypes in this study population. According to the highest silhouette median value (Supplemental Figure S1), four different clusters were identified (Figure 3), named A, B, C, and D. Interestingly, PEs occurrence was significantly different among clusters (*p =* .007). Furthermore, neither BMI nor gender was statistically significant across the clusters (Table 4). Likewise, depression status and social class were not significantly different across the clusters (*p >* .05 in both variables, data not shown). Further examination of the clusters revealed that cluster D exhibited a high probability of developing PEs. This cluster exhibited a PEs occurrence of 71%, while clusters A, B, and C showed a PEs occurrence of 42%, 29%, and 19%, respectively.

**Table 4:** Descriptive Data of the ALSPAC Individuals by Cluster

	Cluster A	Cluster B	Cluster C	Cluster D	*p*
PEs, Cases/Controls, *n*	14/19	4/10	5/21	12/5	.007
Male/Female, *n*	17/16	8/6	13/13	11/6	.781
BMI, kg/m^2^, Mean ± SD	17.43 ± 2.29	17.95 ± 3.51	18.88 ± 2.68	17.33 ± 2.72	.170

Descriptive information was compared between clusters. Statistical comparisons are from Pearson chi-square or Student’s *t* test as appropriate.

ALSPAC, Avon Longitudinal Study of Parents and Children; BMI, body mass index; PEs, psychotic experiences.

Clusters were then examined for associations between the cholesterol data with the resulting KODAMA scores. In total, nine cholesterol parameters (different parameters related to low-density lipoprotein [LDL], very low-density lipoprotein, and intermediate-density lipoprotein with specific particle sizes) were significantly associated with the clustering (Supplemental Table S3). Similarly, KODAMA score plots were performed (Supplemental Figure S3), colored by the resulted clusters, PEs occurrence, gender, and BMI. Score plots color coded by the concentration of small LDL particles and the phospholipids to total lipids ratio in small LDL particles were also performed for visualization and interpretation purposes. There was a significant difference in distribution of PEs cases across the clusters (Supplemental Figure S3B). Interestingly, the levels of certain lipoproteins across the clusters were also statistically different (Supplemental Table S3). Of particular note were differences in the small LDL particles and phospholipid to total lipid ratio in small LDL particles, with a similar distribution to PEs cases. Additional cholesterol-related parameters are shown in Supplemental Figure S4. In summary, cluster D represented a metabolic phenotype with a high probability of developing PE.

## Discussion

The present findings point to early dysregulation of both the lipidome and proteome several years before the development of PEs. Our findings are relevant to PD, anxiety disorder, and depression, as approximately 20% to 30% of subjects who experience PEs go on to develop PD (52), with approximately 50% to 60% going on to develop other psychiatric comorbid disorders (2). The present findings support the literature that phospholipid metabolism and the proteins of the coagulation cascade are abnormal in schizophrenia and depression 26, 53, 54, 55 and extend this literature by providing evidence for such alterations in early childhood before the development of PEs. Furthermore, the present findings are broadly in line with our findings from the previous discovery metabolomics, lipidomic, and proteomic study in the ALSPAC cohort, in which we demonstrated similar changes at 12 years of age for subjects who later went on to develop PD (22). The findings have the potential to contribute to risk calculators for future psychotic illness and mental disorders 4, 56, 57 as well as to an increased understanding of psychosis and psychiatric illness as a multisystem disorder involving lipids and proteins 22, 23, 29. Critically, a novelty of our study lies in the integration of proteomic and lipidomic data, specifically of the PCs and LPCs and the protein members of the complement and coagulation cascades from the same subjects. In so doing, we have identified a robust yet unexpected interdependence of these biological processes that underpin psychotic disease. A tangible advance derived is that our findings highlight early lipid and protein changes associated with vulnerability to a broad range of PD and, in so doing, identify potential novel therapeutic targets.

There is no simple interpretation of the findings of early LPC and PC changes in relation to later psychiatric diseases. However, it is noteworthy that several lines of evidence implicate altered LPC and PC levels in early life and medical morbidities in later life (58). First, Hellmuth *et al.* (59) observed a positive correlation between LPCs in cord blood during pregnancy and early weight gain and later-life high BMI. Second, Rzehak *et al.* (60) showed that LPC(14:0) and PC(38:3) measured at 6 months of age positively correlated with overweight/obesity at 6 years of age. Similar to our findings, these observations suggest an early metabolic alteration that can trigger later disorder (60). Third, a cross-sectional study of early life suggested an inverse association between obesity and LPC(18:1), LPC(18:2), and LPC(20:4) in obese individuals between 6 and 15 years of age (61). These LPCs were also found at lower levels in obese children between 7 and 15 years of age in another cross-sectional study (62). Fourth, an investigation of adults sampled in the Western Australian Pregnancy Cohort showed decreased LPC(18:2) and LPC(18:1) levels in obese subjects compared with normal-weight individuals independent of LDL and high-density lipoprotein cholesterol levels, while LPC(14:0) and PC(32:2) were positively correlated with homeostatic model assessment of insulin resistance, as a measure of insulin resistance, in the same study (63). Overall, these studies suggest elevation of certain LPCs preceding later metabolic disorder and PD.

Perry *et al.* (29) recently showed an association between insulin resistance at 9 years of age and PEs at 18 years of age in the ALSPAC birth cohort. Insulin resistance was also associated with inflammation markers suggesting that inflammation and metabolic risk factors interact to increase risk of psychosis in some people (29). In relation to this, although opposite effects have also been reported 64, 65, reduced levels of specific LPCs have been connected with insulin resistance (45), impaired glucose tolerance (66), and progression to diabetes (67). Furthermore, schizophrenia has been associated with a high prevalence of other comorbid disorders such as diabetes (68), metabolic syndrome (69), and cardiovascular disease (70). Therefore, the early biomarkers such as LPC(18:2), PC(34:2), and PC(32:1) found in the present study may reflect a shared vulnerability to both psychosis and cardiometabolic disorders 58, 67, 71. Previous lipidomic studies in psychosis have identified elevated plasma levels of LPC(16:0), LPC(18:0), LPC(18:1), and LPC(18:2) in first-episode neuroleptic drug-naïve schizophrenia patients as compared with healthy control subjects (72). However, there are inconsistencies in the reported literature, with one study reporting diminished levels of LPCs in the serum of schizophrenia patients compared with their co-twins as well as healthy control subjects (16).

Both the coagulation and the complement pathways have recently been highlighted in schizophrenia 57, 73, 74. Our current study used the semitargeted proteomic method of DIA to extend these findings and show that upregulation of PLG within the coagulation pathway at 12 years of age is associated with later PEs. This more complete analysis of the coagulation pathway proteins in PEs was then combined with complement pathway protein data already available to us on the same subjects (42) to allow a unique integration of lipidomic, complement, and coagulation data. Our integrative network analysis demonstrates that PLG had the strongest connections to PCs and LPCs that were increased in the PEs group. The role of PLG as a carrier for PCs and LPCs was previously investigated by Edelstein *et al.* (75), who suggested that oxidized PCs are integral components of circulating PLG, and Leibundgut *et al.* (76), who showed that PLG covalently binds oxidized phospholipids that influence fibrinolysis, which has known roles associated with neuroinflammation and neurodegeneration (77). Therefore, increased PLG such as we described in PEs is very consistent with higher specific PC and LPC concentrations in the PEs group. Our findings of elevated levels of PLG in subjects who later report PEs are intriguing in light of recent evidence that blood-derived PLG drives brain inflammation (78) and evidence that alpha2-antiplasmin, which is the main inhibitor of PLG-derived plasmin, is upregulated in schizophrenia (79). Interestingly, proteomics studies discovered a high number of complement and coagulation proteins as lipoprotein-associated components, such as complement 4A, complement C4B, vitronectin, clusterin, complement factor H, alpha1/2-antiplasmin, and kininogen, among others (80). There is a surprisingly strong overlap with the proteins that correlate with phospholipids in this study and those that are upregulated in schizophrenia (11). Together, the data provide a link among phospholipid binding proteins, (apo)lipoproteins, complement, and coagulation, and they support growing literature implicating these processes in neuroinflammation and neurodegeneration 77, 81.

Schizophrenia may represent an etiologically heterogeneous disorder, with some subjects having a largely inflammatory basis and some an autoimmune etiology 23, 82, 83. Similarly, it is appreciated that there are heterogeneous outcomes among subjects who experience PEs (2). This may have relevance to the results of KODAMA (36) analysis in which we identified four main clusters, of which cluster D was associated with a high probability of subjects within that cluster experiencing PEs. Interestingly, the lipoprotein particle size parameters were also significantly different across the clusters, with cluster D having increased levels of small LDL particles. Smaller LDL particles are more susceptible to oxidation than larger particles, being more frequently associated to metabolic diseases 84, 85, 86. However, in the present study, the oxidation status and lipidomic analyses on specific LDL particle size were not included at 12 years of age, and thus the results should be interpreted with caution. Future studies evaluating different LDL subtypes might clarify these observed associations.

The present study has several strengths: the longitudinal ALSPAC cohort was used and included both longitudinal clinical assessments and biosampling. The use of samples before disease onset rules out the potential confounding from medications. Furthermore, in contrast to most other studies, our study focused on children who were well at the time of biosampling, unlike other studies, in which the subjects already had experienced a first episode of psychosis. The multiomics integration has allowed a unique insight into the existence of a functional relationship between these lipids and proteins that was unknown previously in the context of psychosis. Future work may look at the broader relationship between proteome and lipidome beyond those specific compounds that we described as discriminant for PEs prediction in this study. A number of limitations should also be acknowledged. First, the lack of validation in a similar cohort of subjects with PEs is a limitation. Second, while depletion of high-abundance proteins did not impact PLG, three of the 22 proteins had been depleted, so they were interpreted with caution. We did not covary for depression, as depression can be considered a transdiagnostic comorbidity, and thus our findings are not necessarily specific to PEs. This is reasonable, as PEs are accepted to represent a vulnerability to a broad range of psychiatric illnesses (2).

### Conclusions

Our study provides evidence for protein and lipid signatures at 12 years of age in subjects who are apparently well but who report PEs at 18 years of age. These changes are not necessarily specific to PEs, as overlapping changes have been observed previously at 12 years of age in subjects who later develop PD (22) and are also observed in association with prediabetes and obesity, and before other cardiometabolic disorders 61, 63, 70, suggesting that these disorders share aspects of their developmental origins. Although there are inconsistences in the literature in terms of metabolic disorders and schizophrenia 24, 87, the present study strongly suggests that there is early vulnerability to the development of PEs and that this involves molecular interconnections between the lipidome and the proteome.

## References

[1] Larsen T.K., Melle I., Auestad B., Haahr U., Joa I., Johannessen J.O. (2011). Early detection of psychosis: Positive effects on 5-year outcome. Psychol Med.

[2] Rutigliano G., Valmaggia L., Landi P., Frascarelli M., Cappucciati M., Sear V. (2016). Persistence or recurrence of non-psychotic comorbid mental disorders associated with 6-year poor functional outcomes in patients at ultra high risk for psychosis. J Affect Disord.

[3] Amminger G.P., Schäfer M.R., Papageorgiou K., Klier C.M., Cotton S.M., Harrigan S.M. (2010). Long-chain ω-3 fatty acids for indicated prevention of psychotic disorders. Arch Gen Psychiatry.

[4] Clark S.R., Baune B.T., Schubert K.O., Lavoie S., Smesny S., Rice S.M. (2016). Prediction of transition from ultra-high risk to first-episode psychosis using a probabilistic model combining history, clinical assessment and fatty-acid biomarkers. Transl Psychiatry.

[5] Kelleher I., Connor D., Clarke M.C., Devlin N., Harley M., Cannon M. (2012). Prevalence of psychotic symptoms in childhood and adolescence: A systematic review and meta-analysis of population-based studies. Psychol Med.

[6] Linscott R.J., van Os J. (2013). An updated and conservative systematic review and meta-analysis of epidemiological evidence on psychotic experiences in children and adults: On the pathway from proneness to persistence to dimensional expression across mental disorders. Psychol Med.

[7] Welham J., Scott J., Williams G., Najman J., Bor W., O’Callaghan M., McGrath J. (2009). Emotional and behavioural antecedents of young adults who screen positive for non-affective psychosis: A 21-year birth cohort study. Psychol Med.

[8] Poulton R., Caspi A., Moffitt T.E., Cannon M., Murray R., Harrington H. (2000). Children’s self-reported psychotic symptoms and adult schizophreniform disorder: A 15-year longitudinal study. Arch Gen Psychiatry.

[9] McGrath J.J., Saha S., Al-Hamzawi A., Andrade L., Benjet C., Bromet E.J. (2016). The bidirectional associations between psychotic experiences and DSM-IV mental disorders. Am J Psychiatry.

[10] Kelleher I., Keeley H., Corcoran P., Lynch F., Fitzpatrick C., Devlin N. (2012). Clinicopathological significance of psychotic experiences in non-psychotic young people: Evidence from four population-based studies. Br J Psychiatry.

[11] Yang J., Chen T., Sun L., Zhao Z., Qi X., Zhou K. (2011). Potential metabolite markers of schizophrenia. Mol Psychiatry.

[12] van Os J., Kapur S. (2009). Schizophrenia. Lancet.

[13] Orešič M., Tang J., Seppänen-Laakso T., Mattila I., Saarni S.E., Saarni S.I. (2011). Metabolome in schizophrenia and other psychotic disorders: A general population-based study. Genome Med.

[14] Holmes E., Tsang T.M., Huang J.T.-J., Leweke F.M., Koethe D., Gerth C.W. (2006). Metabolic profiling of CSF: Evidence that early intervention may impact on disease progression and outcome in schizophrenia. PLoS Med.

[15] Schwarz E., Prabakaran S., Whitfield P., Major H., Leweke F.M., Koethe D. (2008). High throughput lipidomic profiling of schizophrenia and bipolar disorder brain tissue reveals alterations of free fatty acids, phosphatidylcholines, and ceramides. J Proteome Res.

[16] Orešič M., Seppänen-Laakso T., Sun D., Tang J., Therman S., Viehman R. (2012). Phospholipids and insulin resistance in psychosis: A lipidomics study of twin pairs discordant for schizophrenia. Genome Med.

[17] Schneider M., Levant B., Reichel M., Gulbins E., Kornhuber J., Müller C.P. (2017). Lipids in psychiatric disorders and preventive medicine. Neurosci Biobehav Rev.

[18] Steen V.M., Skrede S., Polushina T., López M., Andreassen O.A., Fernø J., Hellard S Le (2017). Genetic evidence for a role of the SREBP transcription system and lipid biosynthesis in schizophrenia and antipsychotic treatment. Eur Neuropsychopharmacol.

[19] Prabakaran S., Swatton J.E., Ryan M.M., Huffaker S.J., Huang J.-J., Griffin J.L. (2004). Mitochondrial dysfunction in schizophrenia: Evidence for compromised brain metabolism and oxidative stress. Mol Psychiatry.

[20] Yao J.K., Dougherty G.G., Reddy R.D., Keshavan M.S., Montrose D.M., Matson W.R. (2010). Altered interactions of tryptophan metabolites in first-episode neuroleptic-naive patients with schizophrenia. Mol Psychiatry.

[21] Rice S.M., Schäfer M.R., Klier C., Mossaheb N., Vijayakumar N., Amminger G.P. (2015). Erythrocyte polyunsaturated fatty acid levels in young people at ultra-high risk for psychotic disorder and healthy adolescent controls. Psychiatry Res.

[22] O’Gorman A., Suvitaival T., Ahonen L., Cannon M., Zammit S., Lewis G. (2017). Identification of a plasma signature of psychotic disorder in children and adolescents from the Avon Longitudinal Study of Parents and Children (ALSPAC) cohort. Transl Psychiatry.

[23] English J.A., Lopez L.M., O’Gorman A., Focking M., Hryniewiecka M., Scaife C. (2018). Blood-based protein changes in childhood are associated with increased risk for later psychotic disorder: Evidence from a nested case-control study of the ALSPAC longitudinal birth cohort. Schizophr Bull.

[24] Davison J., O’Gorman A., Brennan L., Cotter D.R. (2018). A systematic review of metabolite biomarkers of schizophrenia. Schizophr Res.

[25] Bošković M., Vovk T., Kores Plesničar B., Grabnar I. (2011). Oxidative stress in schizophrenia. Curr Neuropharmacol.

[26] Horrobin D.F. (1998). The membrane phospholipid hypothesis as a biochemical basis for the neurodevelopmental concept of schizophrenia. Schizophr Res.

[27] Zammit S., Kounali D., Cannon M., David A.S., Gunnell D., Heron J. (2013). Psychotic experiences and psychotic disorders at age 18 in relation to psychotic experiences at age 12 in a longitudinal population-based cohort study. Am J Psychiatry.

[28] Khandaker G.M., Pearson R.M., Zammit S., Lewis G., Jones P.B. (2014). Association of serum interleukin 6 and C-reactive protein in childhood with depression and psychosis in young adult life. JAMA Psychiatry.

[29] Perry B.I., Upthegrove R., Thompson A., Marwaha S., Zammit S., Singh S.P., Khandaker G. (2019). Dysglycaemia, inflammation and psychosis: Findings from the UK ALSPAC birth cohort. Schizophr Bull.

[30] Boyd A., Golding J., Macleod J., Lawlor D.A., Fraser A., Henderson J. (2013). Cohort profile: The ‘children of the 90s’—the index offspring of the Avon Longitudinal Study of Parents and Children. Int J Epidemiol.

[31] Fraser A., Macdonald-Wallis C., Tilling K., Boyd A., Golding J., Davey Smith G. (2013). Cohort profile: The Avon Longitudinal Study of Parents and Children: ALSPAC mothers cohort. Int J Epidemiol.

[32] World Health Organization, Division of Mental Health (1994). Schedules for clinical assessment in neuropsychiatry: Version 2.

[33] Pluskal T., Castillo S., Villar-Briones A., Orešič M. (2010). MZmine 2: Modular framework for processing, visualizing, and analyzing mass spectrometry-based molecular profile data. BMC Bioinformatics.

[34] Levin Y., Wang L., Schwarz E., Koethe D., Leweke F.M., Bahn S. (2010). Global proteomic profiling reveals altered proteomic signature in schizophrenia serum. Mol Psychiatry.

[35] English J.A., Fan Y., Föcking M., Lopez L.M., Hryniewiecka M., Wynne K. (2015). Reduced protein synthesis in schizophrenia patient-derived olfactory cells. Transl Psychiatry.

[36] Cacciatore S., Tenori L., Luchinat C., Bennett P.R., MacIntyre D.A. (2017). KODAMA: An R package for knowledge discovery and data mining. Bioinformatics.

[37] Westerhuis J.A., Hoefsloot H.C.J., Smit S., Vis D.J., Smilde A.K., van Velzen E.J.J. (2008). Assessment of PLSDA cross validation. Metabolomics.

[38] Eriksson L., Jaworska J., Worth A.P., Cronin M.T.D., McDowell R.M., Gramatica P. (2003). Methods for reliability and uncertainty assessment and for applicability evaluations of classification- and regression-based QSARs. Environ Health Perspect.

[39] Madrid-Gambin F., Garcia-Aloy M., Vázquez-Fresno R., Vegas-Lozano E., de Villa Jubany M.C.R., Misawa K. (2016). Impact of chlorogenic acids from coffee on urine metabolome in healthy human subjects. Food Res Int.

[40] Rohart F., Gautier B., Singh A., Lê Cao K.-A. (2017). mixOmics: An R package for ’omics feature selection and multiple data integration. PLoS Comput Biol.

[41] Moyon T., Le Marec F., Qannari E.M., Vigneau E., Le Plain A., Courant F. (2012). Statistical strategies for relating metabolomics and proteomics data: A real case study in nutrition research area. Metabolomics.

[42] Föcking M., Sabherwal S., Cates H.M., Scaife C., Dicker P., Hryniewiecka M. (2019). Complement pathway changes at age 12 are associated with psychotic experiences at age 18 in a longitudinal population-based study: Evidence for a role of stress. Mol Psychiatry.

[43] González I., Cao K.-A.L., Davis M.J., Déjean S. (2012). Visualising associations between paired “omics” data sets. BioData Min.

[44] Bastian M., Heymann S., Jacomy M. (2009). Gephi: An open source software for exploring and manipulating networks. https://gephi.org/publications/gephi-bastian-feb09.pdf.

[45] Wallace M., Morris C., O’Grada C.M., Ryan M., Dillon E.T., Coleman E. (2014). Relationship between the lipidome, inflammatory markers and insulin resistance. Mol BioSyst.

[46] Cacciatore S., Luchinat C., Tenori L. (2014). Knowledge discovery by accuracy maximization. Proc Natl Acad Sci U S A.

[47] Reynolds A.P., Richards G., de la Iglesia B., Rayward-Smith V.J. (2006). Clustering rules: A comparison of partitioning and hierarchical clustering algorithms. J Math Model Algorithms.

[48] Rousseeuw P.J. (1987). Silhouettes: A graphical aid to the interpretation and validation of cluster analysis. J Comput Appl Math.

[49] Bray R., Cacciatore S., Jiménez B., Cartwright R., Digesu A., Fernando R. (2017). Urinary metabolic phenotyping of women with lower urinary tract symptoms. J Proteome Res.

[50] Cameron A.C., Windmeijer F.A.G. (1997). An R-squared measure of goodness of fit for some common nonlinear regression models. J Econom.

[51] Drenos F., Davey Smith G., Ala-Korpela M., Kettunen J., Würtz P., Soininen P. (2016). Metabolic characterization of a rare genetic variation within APOC3 and its lipoprotein lipase-independent effects. Circ Cardiovasc Genet.

[52] Fusar-Poli P., Bonoldi I., Yung A.R., Borgwardt S., Kempton M.J., Valmaggia L. (2012). Predicting psychosis. Arch Gen Psychiatry.

[53] Khan M.M., Evans D.R., Gunna V., Scheffer R.E., Parikh V.V., Mahadik S.P. (2002). Reduced erythrocyte membrane essential fatty acids and increased lipid peroxides in schizophrenia at the never-medicated first-episode of psychosis and after years of treatment with antipsychotics. Schizophr Res.

[54] Pawełczyk T., Grancow M., Kotlicka-Antczak M., Trafalska E., Gębski P., Szemraj J. (2015). Omega-3 fatty acids in first-episode schizophrenia - a randomized controlled study of efficacy and relapse prevention (OFFER): Rationale, design, and methods. BMC Psychiatry.

[55] Liu X., Li J., Zheng P., Zhao X., Zhou C., Hu C. (2016). Plasma lipidomics reveals potential lipid markers of major depressive disorder. Anal Bioanal Chem.

[56] Cannon T.D., Yu C., Addington J., Bearden C.E., Cadenhead K.S., Cornblatt B.A. (2016). An individualized risk calculator for research in prodromal psychosis. Am J Psychiatry.

[57] Jeffries C.D., Perkins D.O., Fournier M., Do K.Q., Cuenod M., Khadimallah I. (2018). Networks of blood proteins in the neuroimmunology of schizophrenia. Transl Psychiatry.

[58] Rauschert S., Kirchberg F.F., Marchioro L., Koletzko B., Hellmuth C., Uhl O. (2017). Early programming of obesity throughout the life course: A metabolomics perspective. Ann Nutr Metab.

[59] Hellmuth C., Uhl O., Standl M., Demmelmair H., Heinrich J., Koletzko B., Thiering E. (2017). Cord blood metabolome is highly associated with birth weight, but less predictive for later weight development. Obes Facts.

[60] Rzehak P., Hellmuth C., Uhl O., Kirchberg F.F., Peissner W., Harder U. (2014). Rapid growth and childhood obesity are strongly associated with lysoPC(14:0). Ann Nutr Metab.

[61] Wahl S., Yu Z., Kleber M., Singmann P., Holzapfel C., He Y. (2012). Childhood obesity is associated with changes in the serum metabolite profile. Obes Facts.

[62] Butte N.F., Liu Y., Zakeri I.F., Mohney R.P., Mehta N., Voruganti V.S. (2015). Global metabolomic profiling targeting childhood obesity in the Hispanic population. Am J Clin Nutr.

[63] Rauschert S., Uhl O., Koletzko B., Kirchberg F., Mori T.A., Huang R.-C. (2016). Lipidomics reveals associations of phospholipids with obesity and insulin resistance in young adults. J Clin Endocrinol Metab.

[64] Shi A.-H., Yoshinari M., Wakisaka M., Iwase M., Fujishima M. (1999). Lysophosphatidylcholine molecular species in low density lipoprotein of type 2 diabetes. Horm Metab Res.

[65] Hashimoto T., Imamura M., Etoh T., Sekiguchi N., Masakado M., Inoguchi T. (2002). Lysophosphatidylcholine inhibits the expression of prostacyclin stimulating factor in cultured vascular smooth muscle cells. J Diabetes Complications.

[66] Wang-Sattler R., Yu Z., Herder C., Messias A.C., Floegel A., He Y. (2012). Novel biomarkers for pre-diabetes identified by metabolomics. Mol Syst Biol.

[67] Suvitaival T., Bondia-Pons I., Yetukuri L., Pöhö P., Nolan J.J., Hyötyläinen T. (2018). Lipidome as a predictive tool in progression to type 2 diabetes in Finnish men. Metabolism.

[68] Bortolasci C.C., Berk M., Walder K. (2017). First-episode schizophrenia and diabetes risk. JAMA Psychiatry.

[69] Vancampfort D., Stubbs B., Mitchell A.J., De Hert M., Wampers M., Ward P.B. (2015). Risk of metabolic syndrome and its components in people with schizophrenia and related psychotic disorders, bipolar disorder and major depressive disorder: A systematic review and meta-analysis. World Psychiatry.

[70] Westman J., Eriksson S.V., Gissler M., Hällgren J., Prieto M.L., Bobo W.V. (2018). Increased cardiovascular mortality in people with schizophrenia: A 24-year national register study. Epidemiol Psychiatr Sci.

[71] Floegel A., Kühn T., Sookthai D., Johnson T., Prehn C., Rolle-Kampczyk U. (2018). Serum metabolites and risk of myocardial infarction and ischemic stroke: A targeted metabolomic approach in two German prospective cohorts. Eur J Epidemiol.

[72] Cai H.-L., Li H.-D., Yan X.-Z., Sun B., Zhang Q., Yan M. (2012). Metabolomic analysis of biochemical changes in the plasma and urine of first-episode neuroleptic-naïve schizophrenia patients after treatment with risperidone. J Proteome Res.

[73] Sekar A., Bialas A.R., de Rivera H., Davis A., Hammond T.R., Kamitaki N. (2016). Schizophrenia risk from complex variation of complement component 4. Nature.

[74] Hoirisch-Clapauch S., Amaral O.B., Mezzasalma M.A.U., Panizzutti R., Nardi A.E. (2016). Dysfunction in the coagulation system and schizophrenia. Transl Psychiatry.

[75] Edelstein C., Pfaffinger D., Yang M., Hill J.S., Scanu A.M. (2010). Naturally occurring human plasminogen, like genetically related apolipoprotein(a), contains oxidized phosphatidylcholine adducts. Biochim Biophys Acta.

[76] Leibundgut G., Arai K., Orsoni A., Yin H., Scipione C., Miller E.R. (2012). Oxidized phospholipids are present on plasminogen, affect fibrinolysis, and increase following acute myocardial infarction. J Am Coll Cardiol.

[77] Ryu J.K., Rafalski V.A., Meyer-Franke A., Adams R.A., Poda S.B., Rios Coronado P.E. (2018). Fibrin-targeting immunotherapy protects against neuroinflammation and neurodegeneration. Nat Immunol.

[78] Baker S.K., Chen Z.-L., Norris E.H., Revenko A.S., MacLeod A.R., Strickland S. (2018). Blood-derived plasminogen drives brain inflammation and plaque deposition in a mouse model of Alzheimer’s disease. Proc Natl Acad Sci U S A.

[79] Cooper J.D., Ozcan S., Gardner R.M., Rustogi N., Wicks S., van Rees G.F. (2017). Schizophrenia-risk and urban birth are associated with proteomic changes in neonatal dried blood spots. Transl Psychiatry.

[80] von Zychlinski A., Kleffmann T. (2015). Dissecting the proteome of lipoproteins: New biomarkers for cardiovascular diseases?. Transl Proteomics.

[81] Hong S., Beja-Glasser V.F., Nfonoyim B.M., Frouin A., Li S., Ramakrishnan S. (2016). Complement and microglia mediate early synapse loss in Alzheimer mouse models. Science.

[82] Barry H., Hardiman O., Healy D.G., Keogan M., Moroney J., Molnar P.P. (2011). Anti-NMDA receptor encephalitis: An important differential diagnosis in psychosis. Br J Psychiatry.

[83] Fillman S.G., Sinclair D., Fung S.J., Webster M.J., Shannon Weickert C. (2014). Markers of inflammation and stress distinguish subsets of individuals with schizophrenia and bipolar disorder. Transl Psychiatry.

[84] Sigurdardottir V., Fagerberg B., Hulthe J. (2002). Circulating oxidized low-density lipoprotein (LDL) is associated with risk factors of the metabolic syndrome and LDL size in clinically healthy 58-year-old men (AIR study). J Intern Med.

[85] Austin M.A. (1992). Genetic epidemiology of low-density lipoprotein subclass phenotypes. Ann Med.

[86] Ramasamy I. (2018). Update on the laboratory investigation of dyslipidemias. Clin Chim Acta.

[87] McEvoy J., Baillie R.A., Zhu H., Buckley P., Keshavan M.S., Nasrallah H.A. (2013). Lipidomics reveals early metabolic changes in subjects with schizophrenia: Effects of atypical antipsychotics. PLoS One.

