# Supplementary material for: Integrated Lipidomics and Proteomics Point to Early Blood-Based Changes in Childhood Preceding Later Development of Psychotic Experiences: Evidence From the Avon Longitudinal Study of Parents and Children
Source: Biol Psychiatry. 2019 Jul 1;86(1):25–34. doi: 10.1016/j.biopsych.2019.01.018 (PMC6579334; doi:10.1016/j.biopsych.2019.01.018)
Supplement: Supplemental Material [file mmc1.pdf]

Integrated Lipidomics and Proteomics Point to Early Blood-based Changes in Childhood Preceding Later Development of Psychotic Experiences: Evidence From the Avon Longitudinal Study of Parents and Children

Supplemental Information

Lipidomic Analysis and Data Preprocessing

Lipidomic data were firstly processed using MZmine 2 (1), then normalized by lipid-class specific internal standards, and quantified using the inverse-weighted linear model. Signals of internal standards were identified from the standards runs. The internal MS library of retention times was adapted to the study by means of a linear correction based on the observed retention times in the standards runs using the open source R software (2).

The dataset was filtered, allowing the signal to be missing in a maximum of 50 % of the samples at each of the eight batches. Missing values that remained were then imputed with feature-wise half-the-minimum. All lipids that were present in more than 75% of samples were considered for statistical analyses.

High-Abundance Protein Depletion of Human Plasma Samples

To improve the dynamic range for proteomic analysis, 40µl of plasma from each case was immunodepleted for removal of the 14 most abundant proteins (Alpha-1-antitrypsin, A1-acid glycoprotein, Serum Albumin, Alpha2-macroglobulin, Apolipoprotein A-I, Apolipoprotein A-II, Complement C3, Fibrinogen alpha/beta/gamma, Haptoglobin, IgG A, IgG G, IgG M, Transthyretin, and Serotransferrin) using the Agilent Hu14 Affinity Removal System (MARS) coupled to a High Performance Liquid Chromatography (HPLC, Shimadzu LC-10AT) system (3). Protein depletion was undertaken according to the manufacturer's instructions and buffer exchange was performed with 50mM ammonium bicarbonate using spin columns with a 10kDA-molecular weight cut-off (Merck Millipore). Prior to sample preparation for mass

spectrometry, the protein concentration was determined using a Bradford Assay (4), according to the manufacturer's (BioRad) instructions.

Sample Preparation for Mass Spectrometry

Protein digestion and peptide purification was performed as previously described (5). For quality control (QC), an equal aliquot from each protein digest in the experiment was pooled into one sample for use as an internal QC. This QC standard was injected at the beginning of the MS study to condition the column, and after every ten injections throughout the experiment to monitor the MS performance. To facilitate iRT calculation in Skyline™ for DIA data, protein digests were spiked with the Pierce™ Peptide Retention Time Calibration Mixture (4 fmol/µl), according to the manufacturers' instructions.

Targeted Confirmation of Protein Biomarkers Using Data Independent Acquisition (DIA)

The DIA isolation scheme and multiplexing strategy was based on that from Egertson et al. (2013) in which five 4-*m*/*z* isolation windows are analysed per scan (6, 7). DIA overcomes many of the limitations of untargeted proteomics, for example missing values (8, 9). Samples were run on the Thermo Scientific Q Exactive mass spectrometer in DIA mode. Each DIA cycle contained one full MS–SIM scan and 20 DIA scans covering a mass range of 490–910Th with the following settings: the SIM full scan resolution was 35,000; AGC 1e6; Max IT 55ms; profile mode; DIA scans were set at a resolution of 17,000; AGC target 1e5; Max IT 20ms; loop count 10; MSX count 5; 4.0 m/z isolation windows; centroid mode (6). The cycle time was 2s, which resulted in at least ten scans across the precursor peak. For DIA library generation, QC samples were injected in DDA mode (10) at the beginning of the run, and after every ten injections throughout the run. The relative fragment-ion intensities, peptide-precursor

isotope peaks and retention time of the extracted ion chromatograms from the DIA files were used to confirm the identity of the target molecular species (6, 7).

Preprocessing

For DDA, Label-Free Quantification (LFQ), the human FASTA sequence database was searched with MaxQuant (v1.5.2.8) (11, 12), as described (5). False Discovery rates (FDR's) were set to 1% at the peptide and protein level, and only proteins with at least two peptides (one uniquely assignable to the protein) were considered as reliably identified. LFQ intensity values were used for protein quantification between groups. Only proteins present in >80% of samples in at least one group were taken forward for quantification, and the filtered data was normalised by subtracting the median intensity for each protein.

All DIA data was processed in the open-source Skyline software tool (open-source Skyline software tool (https://skyline.gs.washington.edu)). This tool provided the interface for visual confirmation of protein biomarkers in the samples profiled, without any file conversion. The library was constructed by searching the QC injections, which were interspersed after every ten injections throughout the run, in MaxQuant. As detailed in the online tutorials and publications by the Skyline team, the msms.txt file resulting from the MaxQuant search was used to build the library in Skyline. For our peptide targets, mass chromatograms were extracted for +2 and +3 precursor charge states and their associated fragment ions. Based on our discovery results, we targeted 22 coagulation proteins according to the detailed protocol of Egertson (7). For our dataset, the *m/z* tolerance was < 10 ppm and the average retention time window was 2 minutes. All parent and fragment level data was visually confirmed across the samples run, and peak editing was undertaken where necessary, using the peptide Retention Time (RT), dotproduct (idop), mass accuracy (< 10 ppm), and a confirmed library match to reliably identify and quantify peptides across the DIA runs. For statistical analysis, peak areas

of the fragment level data was filtered from the Skyline document grid for analysis in mapDIA, an open source bioinformatics tool for pre-processing and quantitative analysis of DIA data (13). Total Ion Sum (TIS) intensity normalisation procedure was applied, followed by peptide fragment selection using two standard deviation threshold for outlier detection, in the independent sample setup.

Supplementary Table S1. Coagulation pathway protein names targeted in this study.

Protein names	Gene names	Protein
Plasminogen	PLG	P00747
Antithrombin-III	SERPINC1	P01008
Coagulation factor XI	F11	P03951
Heparin cofactor 2	SERPIND1	P05546
Coagulation factor XIII B chain	F13B	P05160
Coagulation factor IX	F9	P00740
Alpha-2-antiplasmin	SERPINF2	P08697
Kininogen-1	KNG1	P01042
Vitamin D-binding protein	GC	P02774
Alpha-2-macroglobulin	A2M	P01023
Carboxypeptidase B2	CPB2	Q96IY4
Fibrinogen alpha chain	FGA	P02671
Plasma serine protease inhibitor	SERPINA5	P05154
Endothelial protein C receptor	PROCR	Q9UNN8
Coagulation factor VII	F7	P08709
Coagulation factor XIII A chain	F13A1	P00488
von Willebrand factor	VWF	P04275
Coagulation factor V	F5	P12259
Prothrombin	F2	P00734
Coagulation factor XII	F12	P00748
Alpha-1-antitrypsin	SERPINA1	P01009
Coagulation factor X	F10	P00742

Defined by KEGG pathway analysis (http://www.genome.jp/kegg/pathway.html).

Supplementary Table S2. Differential plasma PCs and LPCs between the clusters detected.

Lipid	*P* Value	FDR	LSD Posthoc
LPC(16:0)	7.88E-12	1.45E-11	A - B; D - A; C - B; D - B; D - C
LPC(16:0e)	1.09E-02	1.17E-02	D - A; D - B
LPC(16:0p)	4.37E-02	4.56E-02	D - A; D - B
LPC(16:1)	2.06E-10	3.45E-10	A - B; D - A; C - B; D - B; D - C
LPC(18:0)	3.29E-13	6.96E-13	A - B; D - A; C - B; D - B; D - C
LPC(18:1)*	3.99E-12	7.76E-12	A - B; A - C; D - A; C - B; D - B; D - C
LPC(18:2)*	1.80E-08	2.35E-08	A - B; A - C; D - A; D - B; D - C
LPC(20:3)*	4.41E-09	6.34E-09	A - B; A - C; D - A; D - B; D - C
LPC(20:4)	1.08E-08	1.46E-08	A - B; A - C; D - A; C - B; D - B; D - C
LPC(22:5)	2.38E-02	2.52E-02	D - B; D - C
LPC(22:6)	1.34E-05	1.58E-05	A - B; A - C; D - B; D - C
PC(16:0e/18:1(9Z))	4.87E-23	1.17E-21	A - B; A - C; D - A; C - B; D - B; D - C
PC(30:0)	6.67E-15	1.78E-14	A - B; A - C; D - A; C - B; D - B; D - C
PC(31:0)	4.06E-06	4.96E-06	A - B; D - A; C - B; D - B; D - C
PC(32:0)	1.79E-17	8.58E-17	A - B; A - C; D - A; C - B; D - B; D - C
PC(32:1)	1.04E-09	1.56E-09	A - B; A - C; D - A; C - B; D - B; D - C
PC(32:2)	3.93E-15	1.13E-14	A - B; D - A; C - B; D - B; D - C
PC(33:1)	1.44E-11	2.53E-11	A - B; A - C; D - A; C - B; D - B; D - C
PC(34:1)	1.06E-11	1.90E-11	A - B; A - C; D - A; C - B; D - B; D - C
PC(34:2)*	9.09E-10	1.39E-09	A - B; A - C; D - A; C - B; D - B; D - C
PC(35:1)	9.06E-19	5.02E-18	A - B; A - C; D - A; C - B; D - B; D - C
PC(35:2)	1.29E-14	3.32E-14	A - B; D - A; C - B; D - B; D - C
PC(35:3)	3.02E-12	6.04E-12	A - B; D - A; C - B; D - B; D - C
PC(35:4)	8.15E-10	1.28E-09	A - B; D - A; C - B; D - B; D - C
PC(36:1)	5.71E-18	2.93E-17	A - B; A - C; D - A; C - B; D - B; D - C
PC(36:2)	4.73E-16	1.70E-15	A - B; A - C; D - A; C - B; D - B; D - C
PC(36:2)	3.23E-16	1.23E-15	A - B; D - A; C - B; D - B; D - C
PC(36:4)*	8.19E-19	4.91E-18	A - B; A - C; D - A; C - B; D - B; D - C
PC(36:5)	1.02E-08	1.41E-08	A - B; D - A; C - B; D - B; D - C
PC(37:2)	1.48E-20	1.52E-19	A - B; A - C; D - A; C - B; D - B; D - C
PC(37:3)	1.31E-04	1.48E-04	D - A; D - B; D - C
PC(37:4)	5.73E-05	6.55E-05	A - B; A - C; D - B; D - C
PC(38:2)	2.90E-19	1.90E-18	A - B; A - C; D - A; C - B; D - B; D - C
PC(38:3)	6.42E-15	1.78E-14	A - B; A - C; D - A; C - B; D - B; D - C
PC(38:4)	2.07E-08	2.66E-08	A - B; A - C; D - A; D - B; D - C
PC(38:5)	2.11E-15	6.63E-15	A - B; A - C; D - A; C - B; D - B; D - C
PC(38:6)	2.12E-15	6.63E-15	A - B; A - C; D - A; C - B; D - B; D - C
PC(39:6)	1.36E-05	1.58E-05	A - B; D - A; C - B; D - B; D - C
PC(40:4)	1.98E-16	7.92E-16	A - B; A - C; D - A; C - B; D - B; D - C
PC(40:5)	2.88E-14	7.15E-14	A - B; A - C; D - A; C - B; D - B; D - C
PC(40:5)	5.39E-08	6.81E-08	A - B; A - C; C - B; D - B; D - C
PC(40:6)	1.60E-13	3.60E-13	A - B; A - C; D - A; C - B; D - B; D - C
PC(40:7)	1.45E-19	1.04E-18	A - B; A - C; D - A; C - B; D - B; D - C

Lipid	*P* Value	FDR	LSD Posthoc
PC(40:8)	2.25E-10	3.69E-10	A - B; A - C; D - A; C - B; D - B; D - C
PC(O-22:2/22:3)	2.49E-12	5.13E-12	A - B; A - C; D - A; C - B; D - B; D - C
PC(O-32:1)	2.89E-21	4.16E-20	A - B; A - C; D - A; C - B; D - B; D - C
PC(O-32:0)	1.44E-25	1.04E-23	A - B; A - C; D - A; C - B; D - B; D - C
PC(O-34:2)	2.00E-15	6.63E-15	A - B; A - C; D - A; C - B; D - B; D - C
PC(O-34:3)	2.20E-13	4.80E-13	A - B; A - C; D - A; C - B; D - B; D - C
PC(O-36:2)	1.19E-13	2.76E-13	A - B; A - C; D - A; C - B; D - B; D - C
PC(O-36:3)	2.03E-20	1.83E-19	A - B; A - C; D - A; C - B; D - B; D - C
PC(O-36:4)	3.96E-20	3.17E-19	A - B; A - C; D - A; C - B; D - B; D - C
PC(O-36:5)	1.08E-20	1.30E-19	A - B; A - C; D - A; C - B; D - B; D - C
PC(O-38:4)	5.68E-09	8.02E-09	A - B; A - C; D - A; C - B; D - B; D - C
PC(O-38:5)	2.40E-15	7.20E-15	A - B; A - C; D - A; C - B; D - B; D - C
PC(O-38:5)	7.81E-12	1.45E-11	A - B; D - A; C - B; D - B; D - C
PC(O-38:6)	1.43E-08	1.91E-08	A - B; A - C; D - B; D - C
PC(O-40:5)	5.96E-10	9.53E-10	A - B; A - C; C - B; D - B; D - C
PC(O-40:6)	3.46E-23	1.17E-21	A - B; A - C; D - A; C - B; D - B; D - C
PC(O-42:3)	3.22E-09	4.73E-09	A - B; A - C; D - A; C - B; D - B; D - C
PC(P-18:0/22:6)	1.30E-07	1.62E-07	A - B; A - C; D - A; C - B; D - B; D - C
PC(P-20:0/22:4)	5.27E-17	2.37E-16	A - B; A - C; D - A; C - B; D - B; D - C

Abbreviations: FDR, false discovery rate; LPC, lysophosphatidylcholine; PC, phosphatidylcholine. *P* Value of one-way analysis of variance (ANOVA). Fisher's Least Significant Difference (LSD) method was used as posthoc. Results are listed for the 17 significant compounds. *Increased compounds in agreement with O'Gorman *et al.* that included PD individuals (14).

Supplementary Table S3. Association of KODAMA models with cholesterol parameters at the age of 7.

ALSPAC variable	*P*	FDR
Total cholesterol in medium LDL	<0.001	<0.001
Concentration of small LDL particles	<0.001	<0.001
Total lipids in small LDL	<0.001	<0.001
Phospholipids to total lipids ratio in chylomicrons and extremely large VLDL	<0.001	<0.001
Phospholipids to total lipids ratio in very large VLDL	<0.001	<0.001
Phospholipids in IDL	<0.001	<0.001
Cholesterol esters to total lipids ratio in medium LDL	<0.001	<0.001
Free cholesterol to total lipids ratio in medium LDL	<0.001	<0.001
Phospholipids to total lipids ratio in small LDL	<0.001	<0.001

Abbreviations: IDL, intermediate-density lipoprotein; LDL, low-density lipoprotein; VLDL, very low-density lipoprotein. *P* value of K-test and FDR are shown. Results are classified into demographic data and cholesterol profile. After FDR, PE and 9 cholesterol parameters were related to clustering (FDR-corrected *P*-value < 0.001) for 90 individuals with available cholesterol data at the age of 7.

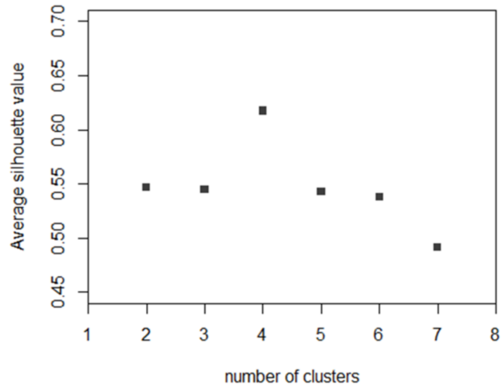

Number of clusters	Average silhouette width of total data set
2	0.546538
3	0.544604
4	0.617278
5	0.54294
6	0.537553
7	0.491248

Supplementary Figure S1. Average silhouette width by number of clusters based on PAM analysis.

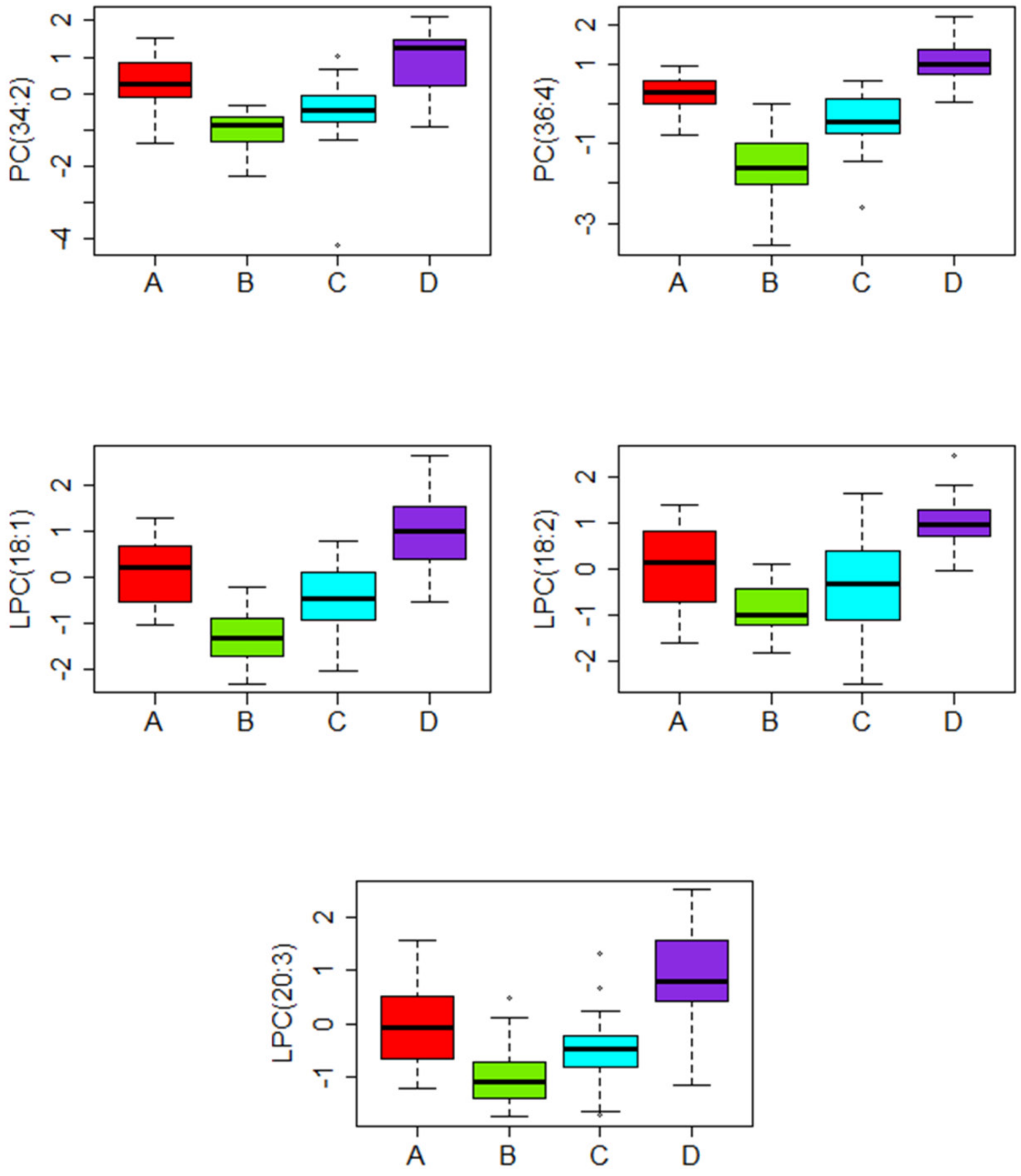

Supplementary Figure S2. Box plots of the distribution of significant lipids among clusters in the present study that are in agreement with O'Gorman 2017 (14). LPC, lysophosphatidylcholine; PC, phosphatidylcholine. Significances of the lipids by clusters are shown in Supplementary Table S1. Cluster D and A was composed by 70.6% and 32.6%, respectively, of PE cases, while cluster B and C was composed by 28.5% and 19.23%, respectively (Chi square p= 0.007).

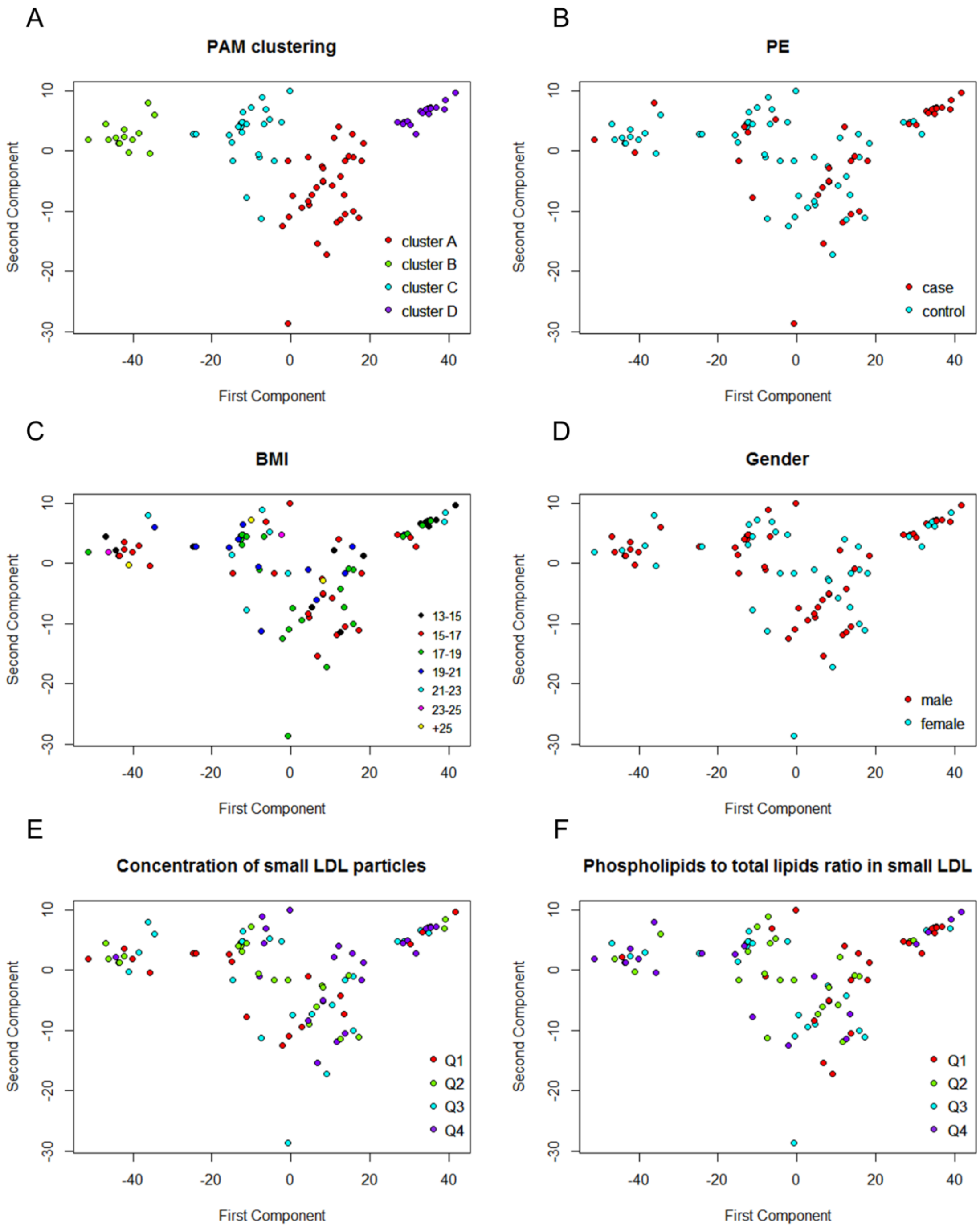

Supplementary Figure S3. Identification of clusters and their relationship with other parameters. KODAMA score plot colored by (A) PAM clustering of KODAMA output colored by clusters; (B) psychotic experiences (K-test FDR-corrected *P*-value = 0.012); (C) body mass index (FDR-corrected *P*-value = 0.405); (D) gender (FDR-corrected p-value = 1.000); (E) concentration of small LDL particles grouped by quantiles (FDR-corrected *P*-value < 0.001) and (F) phospholipids to total lipids ratio in small LDL particles grouped by quantiles (FDR-corrected *P*-value < 0.001).

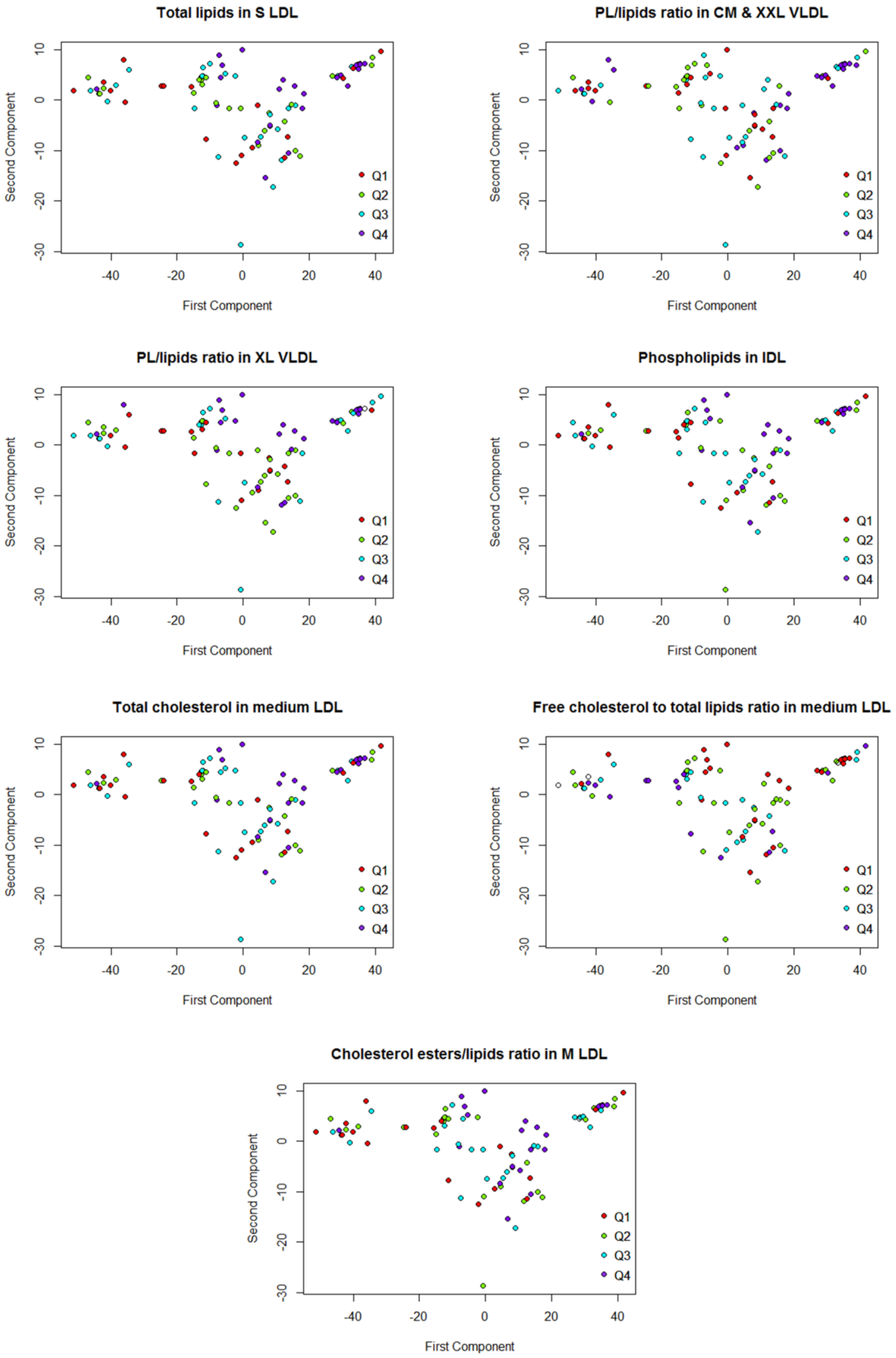

Supplementary Figure S4. KODAMA score plot grouped by quantiles of the significant cholesterol parameters from K-test (FDR-corrected p-value < 0.001). CM, chylomicrons; IDL,

intermediate density lipoprotein particles; L, large; LDL, low density lipoprotein; PL, phospholipids; S, small; VLDL, very low density lipoprotein; XL, very large; XXL, extremely large.

Supplemental References

1. Pluskal T, Castillo S, Villar-Briones A, Orešič M (2010): MZmine 2: Modular framework for processing, visualizing, and analyzing mass spectrometry-based molecular profile data. *BMC Bioinformatics*. 11: 395.

2. R Core Team (2015): R: A language and environment for statistical computing. *R Found Stat Comput*. .

3. Levin Y, Wang L, Schwarz E, Koethe D, Leweke FM, Bahn S (2010): Global proteomic profiling reveals altered proteomic signature in schizophrenia serum. *Mol Psychiatry*. 15: 1088–1100.

4. Bradford MM (1976): A rapid and sensitive method for the quantitation of microgram quantities of protein utilizing the principle of protein-dye binding. *Anal Biochem*. 72: 248–54.

5. English JA, Fan Y, Föcking M, Lopez LM, Hryniewiecka M, Wynne K, *et al.* (2015): Reduced protein synthesis in schizophrenia patient-derived olfactory cells. *Transl Psychiatry*. 5: e663–e663.

6. Egertson JD, Kuehn A, Merrihew GE, Bateman NW, MacLean BX, Ting YS, *et al.* (2013): Multiplexed MS/MS for improved data-independent acquisition. *Nat Methods*. 10: 744–746.

7. Egertson JD, MacLean B, Johnson R, Xuan Y, MacCoss MJ (2015): Multiplexed peptide analysis using data-independent acquisition and Skyline. *Nat Protoc*. 10: 887–903.

8. Sajic T, Liu Y, Aebersold R (2015): Using data-independent, high-resolution mass spectrometry in protein biomarker research: Perspectives and clinical applications. *PROTEOMICS - Clin Appl*. 9: 307–321.

9. Liu Y, Buil A, Collins BC, Gillet LCJ, Blum LC, Cheng L-Y, *et al.* (2015): Quantitative variability of 342 plasma proteins in a human twin population. *Mol Syst Biol*. 11: 786.

10. English JA, Lopez LM, O'Gorman A, Focking M, Hryniewiecka M, Scaife C, *et al.* (2017): Blood-Based Protein Changes in Childhood Are Associated With Increased Risk for Later Psychotic Disorder: Evidence From a Nested Case-Control Study of the ALSPAC Longitudinal Birth Cohort. *Schizophr Bull*. . doi: 10.1093/schbul/sbx075.

11. Cox J, Neuhauser N, Michalski A, Scheltema RA, Olsen J V., Mann M (2011): Andromeda: A Peptide Search Engine Integrated into the MaxQuant Environment. *J Proteome Res*. 10: 1794–1805.

12. Cox J, Mann M (2008): MaxQuant enables high peptide identification rates, individualized p.p.b.-range mass accuracies and proteome-wide protein quantification. *Nat Biotechnol*. 26: 1367–1372.

13. Teo G, Kim S, Tsou C-C, Collins B, Gingras A-C, Nesvizhskii AI, Choi H (2015): mapDIA: Preprocessing and statistical analysis of quantitative proteomics data from data

independent acquisition mass spectrometry. *J Proteomics*. 129: 108–120.

14. O’Gorman A, Suvitaival T, Ahonen L, Cannon M, Zammit S, Lewis G, *et al.* (2017): Identification of a plasma signature of psychotic disorder in children and adolescents from the Avon Longitudinal Study of Parents and Children (ALSPAC) cohort. *Transl Psychiatry*. 7: e1240.
